# Complex Regional Pain Syndrome Type I Following Non-Orthopedic Surgery: Case Report and Narrative Review

**DOI:** 10.3390/diagnostics11091596

**Published:** 2021-09-01

**Authors:** Antimo Moretti, Francesca Gimigliano, Marco Paoletta, Matteo Bertone, Sara Liguori, Giuseppe Toro, Giovanni Iolascon

**Affiliations:** 1 Department of Medical and Surgical Specialties and Dentistry, University of Campania “Luigi Vanvitelli”, 80138 Naples, Italy; antimo.moretti@unicampania.it (A.M.); bertonematteodott@gmail.com (M.B.); sara.liguori@unicampania.it (S.L.); giuseppe.toro@unicampania.it (G.T.); giovanni.iolascon@unicampania.it (G.I.); 2 Department of Mental and Physical Health and Preventive Medicine, University of Campania “Luigi Vanvitelli”, 80138 Naples, Italy; francesca.gimigliano@unicampania.it

**Keywords:** complex regional pain syndromes, reflex sympathetic dystrophy, neridronate, diphosphonates, angioplasty, balloon, coronary

## Abstract

Complex regional pain syndrome type I (CRPS I)—or algodystrophy—is a rare disease that usually occurs after a traumatic event. It is characterized by typical clinical findings such as severe and disabling pain disproportionate to the injury, functional limitations, as well as sensory and vasomotor alterations. However, some people do not report any injury associated with algodystrophy onset in personal history. We describe the management of an unusual case of CRPS I which occurred during the long-term follow-up of percutaneous transluminal coronary angioplasty (PTCA) and performed a narrative review of algodystrophy in non-orthopedic surgery. A clinical case of a 44-year-old man with a spontaneous onset of CRPS I of the right ankle is presented. He did not refer to history of any memorable significant trigger event. Approximately 5 months before the onset of clinical manifestations, he received a PTCA via the right femoral approach. We suppose an association between CRPS and this procedure and propose a possible pathophysiologic mechanism. The patient was treated with intramuscular neridronate, which resulted in significant pain relief and improved his quality of life. A comprehensive clinical and instrumental evaluation in patients with CRPS is challenging but mandatory for a correct diagnosis. An extensive analysis of patient history is important for identifying any potential trigger event, including non-orthopedic procedures. Bone scan could have a pivotal role for improving diagnostic sensitivity and specificity in CRPS I. Neridronate was a safe and effective therapeutic approach for this patient, confirming the results of the high-quality evidence available.

## 1. Introduction

Complex regional pain syndrome type I (CRPS I)—or algodystrophy—is a rare condition that usually occurs a few weeks or months after a traumatic event [1]. It is characterized by typical symptoms and signs that include severe and disabling pain disproportionate to the injury, functional limitation, as well as sensory and vasomotor alterations [2]. The main pathogenic mechanism in the acute phases of CRPS I is an abnormal inflammatory response to injury, probably due to predisposing genetic susceptibility [3,4,5]. For almost all cases, medical history includes a specific triggering factor for the development of CRPS I, such as fractures (>40% of cases) [6,7], sprains, contusions, and surgical procedures. However, some people presenting with this typical clinical scenario that meets the diagnostic criteria (Budapest criteria) [8] have not report any injury associated with algodystrophy onset in personal history. In these cases, it is important to further investigate any risk factors which may trigger pathogenic mechanisms of CRPS I.

We describe the management of an unusual case of CRPS I of the ankle which occurred in the long-term follow-up of percutaneous transluminal coronary angioplasty (PTCA) and formulate a pathophysiological hypothesis according to the available evidence. Moreover, we perform a narrative review about algodystrophy occurring in non-orthopedic surgery.

## 2. Case Presentation

A 44-year-old Caucasian man was referred to our Physical Medicine and Rehabilitation Unit in May 2017 for spontaneous severe pain in the right ankle without any previous trauma. He was 183 cm tall and weighed 85 kg (BMI 24.3 kg/m^2^), an office worker with sedentary lifestyle, former smoker, affected by hypertension, hypercholesterolemia, and myocardial ischemia treated with PTCA via right femoral artery approach in September 2016. At the time, he was taking ramipril (10 mg once a day), atorvastatin (40 mg once a day) and clopidogrel (75 mg once a day).

**Figure 1:**
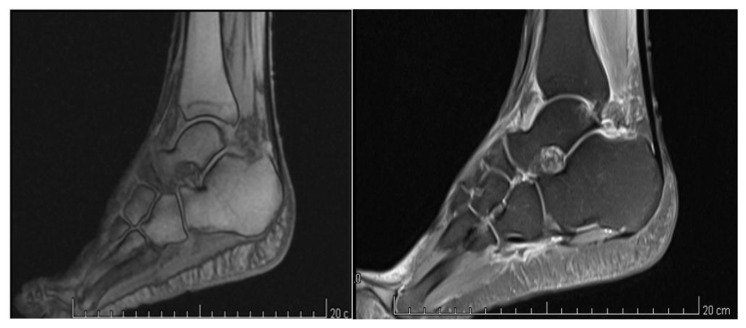
MRI of the right ankle and foot of the patient showing soft tissue edema without bone involvement.

Clinical complaints started in February 2017 with severe continuous pain, worse during the night, in addition to swelling, tenderness, and bruising of his right ankle and foot. For this reason, the patient referred to the emergency room where an ankle X-ray was obtained, but no pathological findings (e.g., fractures) were reported. The patient received a diagnosis of ankle sprain which was managed with the rest, ice, compression, and elevation (RICE) protocol, as well as oral acetaminophen (1000 mg TID for 5 days). Despite the treatment, after 1 week, the symptoms worsened, leading the patient to seek another orthopedic consultation, where he was referred for magnetic resonance imaging (MRI) of the right ankle and foot, which showed diffuse subcutaneous and peritendinous edema, without other significant findings affecting the bones and/or articular cartilages (Figure 1).

**Figure 2:**
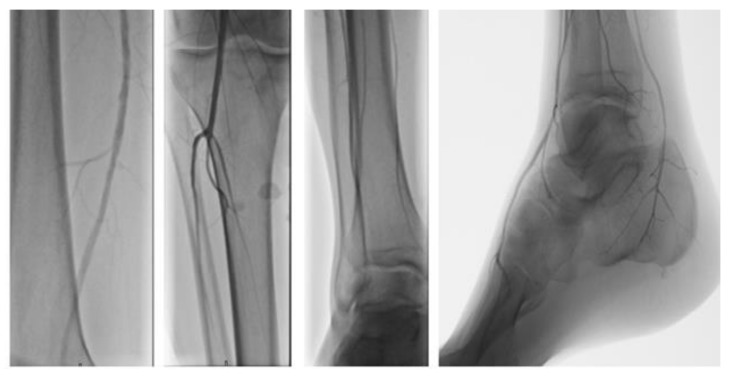
Peripheral vascular angiography showed a normal calibre and patency of the femoral-artery and a good blood supply of the foot.

Assuming a bruise and an inflammatory condition, the patient was prescribed oral ibuprofen, 600 mg bid for 1 week, and local cryotherapy, 20 min for 3 times a day, without symptom remission. A vascular surgeon was also consulted, who requested color Doppler ultrasonography (CDU) and an angiography, which excluded venous thrombosis and peripheral arterial disease (Figure 2).

Finally, the patient came to our attention because the vascular surgeon recommended a physiatric consultation.

**Figure 3:**
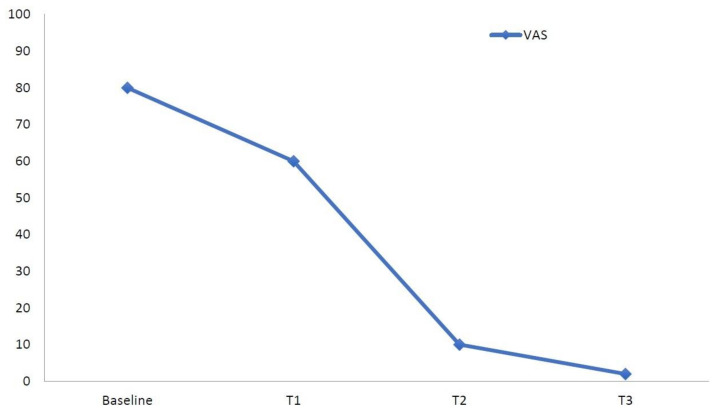
Visual Analogue Scale (VAS) pain trends from baseline to the end of the follow-up in our patient.

**Figure 4:**
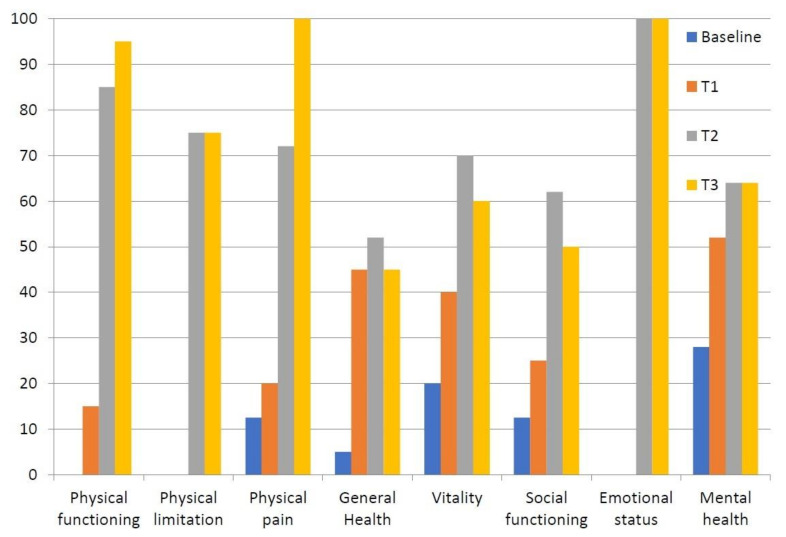
Changes in SF-36 sub-scores of our patient across follow-ups (scores on *Y*-axis, sub-items on *X*-axis).

Upon physical examination, the right ankle and foot appeared swollen and erythematous compared to the contralateral ones. The region was warm and sweaty to the touch, which evoked pain (mechanical allodynia). Moreover, both the active and passive range of motion (ROM) of the ankle were painful with joint stiffness (dorsal flexion 5 degrees, plantar flexion 15 degrees). The Visual Analogue Scale (VAS) for pain was 80/100 mm (Figure 3), whereas the Short-Form McGill Pain Questionnaire (SF-MPQ) [9] and Short Form-36 Health Survey (SF-36) [10] scores were poor (Table 1 and Table 2, Figure 4). The patient was only able to walk with a crutch.

Biochemical assessment did not identify any alterations suggesting rheumatologic or inflammatory disorders. Laboratory exams revealed a normal complete blood count (CBC) and serum levels of erythrocyte sedimentation rate (ESR, 8 mm/h), C-Reactive Protein (CRP, 0.3 mg/L), glucose (72 mg/dL), creatinine (0.96 mg/dL), uric acid (5.0 mg/dL), alkaline phosphatase (80 U/L, normal range 40–129 U/L), calcium (9.4 mg/dL), phosphate (4.3 mg/dL), creatine kinase (68 U/L), and lactic dehydrogenase (177 U/L).

According to the Budapest criteria, this clinical condition suggested the diagnosis of CRPS I.

**Figure 5:**
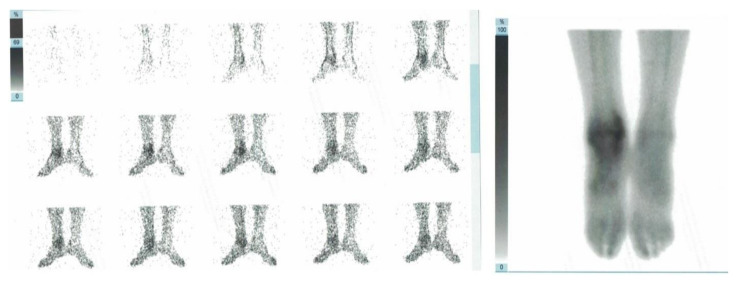
TPBS imaging showing enhanced distribution 3 h after intravenous contrast medium injection involving the symptomatic ankle.

To confirm our diagnosis, we decided to perform a 99mTc-Methyl diphosphonate triphasic bone scan (99mTc-MDP TPBS), which showed an “intense flow associated with enhanced distribution in blood pool phases and osteoblastic activity at 3 h at the distal tibial epiphysis suggestive of CRPS I” (Figure 5).

Therefore, we prescribed daily intramuscular neridronate (25 mg per day for 16 consecutive days, for a total dose of 400 mg), according to the same dosage used through intravenous route in the randomized control trial (RCT) which proved the efficacy of this intervention for algodystrophy [11].

Clinical follow-ups performed at 7 (T1), 30 (T2), and 60 (T3) days after the start of treatment showed a rapid response with significant pain relief preserved over time and an improved health-related quality of life (HRQoL) (Table 1 and Table 2, Figure 3 and Figure 4) without adverse events. Moreover, the patient was able to walk without aids at the end of treatment.

## 3. Discussion

Complex regional pain syndromes are diagnostic and therapeutic dilemmas for clinicians in different settings. Differential diagnosis of these conditions is challenging, particularly for CRPS I, because several diseases are characterized by similar clinical scenarios, such as rheumatic diseases, orthopedic trauma, infections, cardiovascular diseases as well as neurological disorders [12]. From a historical point of view, the countless terms used to name this syndrome over about two centuries [12] are a clue of the extreme complexity in defining the pathophysiological mechanisms resulting in its clinical manifestations. Despite the exponential increase in studies investigating its different issues, from epidemiological analyses to the validity of diagnostic approaches, as well as the therapeutic options proposed, CRPS I remains an unmet need of modern medicine. Indeed, it is not surprising that an interdisciplinary approach is currently the best management strategy for algodystrophy [13].

This condition typically develops after few weeks from an injury with a non-dermatomal pattern involving the distal limb.

In this case report, we describe an unusual form of CRPS I in a patient who received PTCA approximately 5 months before clinical complaints become evident.

Our patient manifested clinical symptoms and signs suggestive of a warm CRPS I [14], including disproportionate pain, allodynia, increased skin temperature, sweating and loss of joint mobility, thus matching the Budapest criteria. To date, this tool is considered the most appropriate and useful for the diagnosis of algodystrophy because of its excellent sensitivity and good specificity [8]. However, the onset of symptoms far from the inciting injury was not reported in these criteria. Several diagnostic tools have been proposed to identify CRPS I [15,16]. Among these, Veldman criteria are the only ones that mentioned the spread of symptoms distally to the injury site, reporting the item “signs and symptoms present in an area larger than the area of primary injury or operation and including the area distal to the primary injury”—as occurred in our case [16].

However, algodystrophy following surgery or other invasive procedures in non-orthopedic settings are rare and often underestimated (Table 3).

For what concerns vascular procedures, Lai et al. described a case of CRPS I of right-hand in a 73-year-old male after a right-side transradial approach for PTCA [17] with signs and symptoms persisting for 3 months after the procedure and treated through non-pharmacological interventions. Parikh et al. [18] reported a case of a 55-year-old man presenting with CRPS of the right foot and ankle after transfemoral coronary balloon angioplasty occurring few weeks after the intervention, and treated with physiotherapy and opioids; however, the authors did not report any detail about the safety and effectiveness of this therapeutic option.

Saad et al. [19] described the case of a 36-year-old Caucasian woman presenting cold-type CRPS of the left foot after transfemoral catheterization for atrioventricular node re-entry tachycardia (AVNRT) ablation and treated with spinal cord stimulator with significant clinical improvements after pharmacological treatment (gabapentin, amitriptyline, vasodilators), physical therapy and sympathetic nerve block which failed to relieve pain and restore functional independence.

Papadimos and Hofmann [20] described the case of a 46-year-old man with CRPS I of the left hand which occurred after transradial cardiac catheterization and the prolonged use of a hemostatic device for the left ventriculography and coronary artery injections and treated by physical therapy with a limited improvement of mobility and pain.

Despite CRPS not usually being triggered by percutaneous vascular procedures, further cases associated with arteriovenous fistula (AVF) [22] and arteriovenous graft [21] have been described. In 1991, Weise and Bernard [21] reported the case of a 58-year-old black woman with CRPS I of the left hand and wrist after approximately 3 months since the placement of a left brachiobasilic arteriovenous graft of polytetrafluorethylene (WIFE) for hemodialysis and treated with prednisone (40 mg/die) with the improvement of signs and symptoms in 6 weeks.

Unek et al. [22] described the case of a 62-year-old patient with CRPS I of the left hand after approximately 1 month since the placement of an AVF between the brachial artery and cephalic vein on the left arm for hemodialysis and treated with calcitonin (400 IU/day) and physical therapy with a reduction in swelling and pain.

Kemler and Tordoir [23] reported three cases of reflex sympathetic dystrophy (RSD) of left hands after thrombosis or the insufficient maturation of fistula in patients who underwent vascular access surgery for hemodialysis; however, the authors did not report any work-up that either treatment provided for these patients.

Cases of CRPS I have also been described following solid organs and bone marrow transplantation (BMT). However, the prevalence of algodystrophy in transplanted patients is poorly known, and many factors could determine its manifestation such as the type of organ transplantation and immunosuppressive therapy. Transplanted patients treated with cyclosporin A (CsA) who develop CRPS I range from 1% to 10% [29]. According to Muñoz-Gomez et al. [24], approximately 3% of patients receiving kidney transplants and treated with CsA developed RSD of lower limbs. In this population, symptoms started after approximately 3 months from surgery with significant improvement after the reduction in CsA dosage, and the mean duration of clinical manifestations were approximately 8 months.

Ybarra et al. [25] described four cases (one man and three women) of RSD of the lower limbs after renal transplantation and immunosuppression with tacrolimus monotherapy or shifted from/to CsA; symptoms spontaneously improved in 3–4 months in two patients and pain gradually resolved in 6 months spontaneously or after prednisone administration in the other two patients.

Stamatoullas et al. [26] reported three cases (two men and one woman) of RSD after BMT, immunosuppressive therapy, chemotherapy, and radiotherapy; two patients were unsuccessfully treated with calcitonin (50 U/day for 5 days), replaced by pamidronate (30 mg/d) for 5 days which resulted effective, while calcitonin (50 U/day for 5 days) improved symptoms in one patient.

Cases of CRPS I were also described in thoracic surgery. According to Graham et al. [27], two women showed algodystrophy of the arm and hand following mastectomy for mastalgia. These patients had no improvements in the symptoms after intensive physiotherapy, intravenous infusions of pamidronate, and despite two stellate ganglion nerve blocks and localized injections of botulinum toxin in one case.

Chen et al. [28] reported the case of a 22-year-old man with CRPS I of the right arm after the Nuss procedure for correcting pectus excavatum, treated with intensive rehabilitation with pain relief and reduction in edema.

Algodystrophy remains a challenging clinical condition to diagnose. In fact, many clinicians did not recognize this syndrome, because even in the patient’s medical history it is not easy to identify any trigger event commonly associated with CRPS. In a large epidemiological study, Ott et al. described that almost all cases of algodystrophy follow fractures, other traumatic injuries, or surgery, but 7% of cases occur without any memorable or harmful event [30].

Our case seems to belong to this category of patients. However, considering that any biological stimulus could provoke CRPS, including invasive procedures, we speculated that coronary angioplasty might be linked to the onset of CRPS. Only one case of CRPS of the ankle associated with transfemoral coronary balloon angioplasty has previously been published [18]. However, neither the potential pathophysiological mechanisms nor a specific therapeutic approach have been described.

In our case, we hypothesize that skin damage during transfemoral coronary angioplasty releases damage-associated molecular products (DAMPs) that stimulate the dendritic cell and its migration to local lymph node [31]. This condition results in the loss of the immune tolerance of lymph node-resident dendritic cell activating adaptive immune response. Inflammatory cytokines release (i.e., TNFα, IL-1, IL-18) and T-cells and B-cells activation result in the production of serum IgG antibodies and inflammatory cascade. In addition, tissue injury induces an autonomic imbalance with increased sympathetic and/or reduced parasympathetic tone. However, described pathophysiological mechanisms do not provide an adequate interpretation of the delayed onset and peripheral symptoms in our case. Usually, warm CRPS lasts no longer than 6 months, later turning into the cold type. In our patient, where warm symptoms started just within this time limit, different mechanisms were probably involved. In patients affected by CRPS, specific serum IgG1 and IgG3 autoantibodies activating β2-adrenergic or muscarinic receptors (M2) were released, causing peripheral vasodilation/constriction [32]. Although a widespread inflammation status was present, we speculate that microcirculatory alteration caused by predominant sympathetic tone may predispose to the development of pain at distal limb. Furthermore, sympathetic activation stimulates peripheral nociceptors in CRPS I. Persistent stimulation of nociceptor might result in the structural modification of these receptors, which in turn prolong their activation. In this regard, a third mechanistic descriptor for chronic pain states has recently been proposed by the International Association for the Study of Pain (IASP) [33]: nociplastic/algopathic/nocipathic pain was described as “Pain that arises from altered nociception despite no clear evidence of actual or threatened tissue damage causing the activation of peripheral nociceptors or evidence for disease or lesion of the somatosensory system causing the pain”. In our case, we speculate that severe chronic pain could be due to this mechanism where the altered modulation of nociceptors caused by plastic structural modification contributes to the persistence of the signs and symptoms. However, skin biopsy with histological analysis to confirm changes of nociceptive nerve fibers was not performed.

Even if patient clinical status was suggestive of CRPS, we decided to perform a 99mTc-MDP TPBS to confirm our hypothesis. A previous MRI showed widespread soft tissue edema involving muscle, synovial tissue, and tendons without the significant bone marrow lesion (BML) of the ankle and foot. In fact, in contrast to what is commonly believed, BML cannot be considered as a typical finding of CPRS or even its synonym. Cappello et al. compared bone scan and MRI for the diagnosis of CRPS I, reporting a significantly higher sensitivity (78.76% vs. 35.33%) and negative predictive value (88% vs. 51.76%) for bone scan [34]. In our case, MRI findings were not suggestive of CRPS, but rather of a rheumatic disorder, while TPBS showed a significant altered signal intensity due to enhanced bone metabolism. The role of bone scintigraphy in CRPS is still debated. Moon et al. suggested that a decreased tracer uptake pattern, also observed during phases I and II of TPBS, could provide additional evidence to Budapest criteria to diagnose CRPS I [35]. Authors highlighted no correlation between the disease onset and TPBS findings, suggesting that the severity of symptoms are not related to clinical stage of algodystrophy. On the other hand, TPBS might have a role for monitoring treatment effectiveness. Patients responding to treatment for CRPS I showed earlier normalization of scintigraphic parameters during early perfusion phase [36]. In our opinion, TPBS should be included in our diagnostic approach of CRPS for better identifying affected patients—regardless of the clinical stages.

Although no drug has been approved by international regulatory agencies (FDA or EMA) for treating algodystrophy, we decided to administer neridronate because this is the only therapy approved by our national regulatory agency (The Italian Medicines Agency, AIFA) for CRPS I [37]. The rationale for the use of bisphosphonates (BPs) consists of their action on several pathophysiological mechanism involved in CRPS. Neridronate seems to influence some immunological phenomena by inhibiting macrophage activation and microenvironmental pro-inflammatory factors involved in pH modulation, thus reducing acidosis, which results from osteoclast activation [38]. Moreover, TPBS showing enhanced bone metabolism justified the use of BPs. This case confirms the benefits of neridronate in CRPS I, with significant and rapid effects in terms of pain relief and improved HRQoL.

## 4. Conclusions

In conclusion, CRPS I remains a challenge for physicians. Several pathophysiological pathways are proposed, but a unanimous consensus has not been reached. The diagnostic process has a pivotal role for starting an appropriate therapeutic approach, particularly considering each potential trigger in the medical history of the patient. Among radiological assessments, bone scintigraphy could be useful for confirming the diagnosis and driving therapeutic decision making. This case confirms the benefits of neridronate in CRPS I, with significant and rapid effects in terms of pain relief and improved HRQoL.

## Figures and Tables

**Table 1:** Change in short form McGill Pain Questionnaire (SF-MPQ) in our case.

Outcome Measure	Baseline (T0)	T1 (1 Week)	T2 (1 Month)	T3 (2 Months)
VAS Pain	80	60	10	0
Sensory subscore	12	7	2	0
Affective subscore	2	2	0	0
Total score	14	9	2	0

**Table 2:** Change in 36-Item Short Form Health Survey (SF-36) assessing the quality of life of our patient.

Outcome Measure (0–100)	Baseline (T0)	T1 (1 Week)	T2 (1 Month)	T3 (2 Months)
Physical functioning	0	15	95	95
Physical limitation	0	0	75	75
Physical pain	12.5	20	72	100
General health	5	45	52	45
Vitality	20	40	70	60
Social functioning	12.5	25	62	50
Emotional status	0	0	100	100
Mental health	28	52	64	64

**Table 3:** Cases of algodystrophy in non-orthopedic surgery.

Authors	Putative Triggering Event	Case Description
Lai C.J. et al. (2006) [17]	Primary percutaneous transluminal coronary angioplasty (PTCA) after myocardial infarction	A 73-year-old male with CRPS I of the right hand. Numbness, pain and soft tissue swelling persisted for 3 months after procedure. Characteristics of CRPS were noted with three-phase bone scan (delayed bone phase). The patient was treated with intensive physical therapy and hydrotherapy, manual soft tissue release and occupational therapy for the hand function, with improvement in range of motion, hand function and activity of daily living.
Parikh R.P. et al. (2016) [18]	Coronary angioplasty with drug-eluting stent via transfemoral artery approach after post-infarct angina	A 55-year-old man with CRPS I of right foot and ankle. Severe burning pain of the dorsum of the foot and ankle, swelling of the ankle joint and allodynia over the ankle joint few weeks after procedure. Triple-phase bone scan showed delayed uptake of tracer in the distal ends of the tibia, fibula, tarsal and metatarsal bones. Patient was treated with physiotherapy and opioid analgesics.
Saad A. et al. (2011) [19]	Transfemoral catheterization-related groin pseudoaneurysm after AV node re-entry tachycardia (AVNRT) ablation	A 36-year-old Caucasian woman with CRPS I of left foot. Numbness, tingling, local edema and mild groin discomfort, swelling and cold sensation after 1 month from procedure. Three-phase bone scan showed cold-type CRPS. Spinal cord stimulator improved symptoms after unsuccessful pharmacological and rehabilitative treatments.
Papadimos T.J. and Hofmann J.P. (2002) [20]	Transradial cardiac catheterization associated with prolonged use of a hemostatic device for the left ventriculography and coronary artery injections after chest pain, bradycardia and a right bundle branch block	A 46-year-old man with CRPS I of left hand. Pallor and pain in the left hand presenting 24 h after discharge; several months later, cold intolerance, burning sensations, paresthesia and loss of left radial pulse accompanied pain, intermittent pallor and limited range of motion. Echo color-Doppler showed the occluded left radial artery for 12 cm proximal to the puncture, but perfusion seemed adequate. The patient was treated with physical therapy but limited range of motion with pain persisted. He refused sympathetic blockade and medications.
Weise W.J. and Bernard D.B. (1991) [21]	Placement of a left brachiobasilic AV graft of polytetrafluorethylene (WIFE) for hemodialysis	A 58-year-old black woman with CRPS I of left hand and wrist. Pain, swelling and reduction in range of motion, erythema, dysesthesia to light touch of left-hand months after approximately 3 months from beginning hemodialysis and placement of a left brachiobasilic AV graft of polytetrafluorethylene (WIFE). X-rays showed diffuse osteopenia and periarticular demineralization of left hand, allowing to make a diagnosis of RSD. The patient was treated with prednisone (40 mg/die) with improvement of symptoms and physical findings in the next 6 weeks.
Unek I.T. et al. (2005) [22]	Placement of an arteriovenous fistula (AVF) between the brachial artery and cephalic vein on left arm for hemodialysis	A 62-year-old patient with CRPS I of the left hand after approximately 1 month of placement AVF. Pain, swelling, cyanosis of the third finger of her left hand and progressive hand pain spreading to other fingers. Physical examination showed impairment of muscle strength (2/5), thenar and hypothenar atrophy, localized edema distal to wrist, flexion contractures on the second and third proximal interphalangeal joints of the left hand. Magnetic resonance angiography showed insufficient flow to distal parts of brachial artery (steal syndrome) at the level of arteriovenous fistula. Bone scan with technetium confirmed diagnosis of RSD at early stage. Patient was treated with calcitonin (400 IU/day) and physical therapy with improvement of swelling and pain.
Kemler M.A. and Tordoir J.H.M. (1998) [23]	Vascular access surgery for hemodialysis and thrombosis or insufficient maturation of fistula	A 71-year-old woman, a 59-year-old woman with RDS of left hand after ischemia for fistula thrombosed and a 70-year-old woman with RDS of left hand after ischemia due to insufficient maturation of the fistula. Loss of hand function, pain, atrophy of skin or subcutaneous tissue. Diagnostic work-up and treatments were not reported.
Muñoz-Gomez J. et al. (1991) [24]	Renal transplantation and immunosuppression with cyclosporin A	Seven patients (five men and two women) with RSD of lower limbs. Severe pain, periarticular soft tissue swelling, articular mobility conserved and walking impairment. Radiography and scintigraphy (99m-technetium methylene diphosphonate) showed osteoporotic pattern and increased uptake 99m-technetium with a periarticular distribution in the clinically affected areas. Symptoms started approximately 3 months after kidney transplantation in all patients and improved reducing cyclosporin A dosage; the mean duration of symptoms was 8 months.
Ybarra J. et al. (2003) [25]	Renal transplantation and immunosuppression with tacrolimus monotherapy or shifted from/to cyclosporin A	Four patients (one man and three women) with RSD of lower limbs. Symptoms and signs (pain, swelling, periarticular soft tissue swelling, difficulty in walking) started between one and four months after immunosuppression or shifted therapy for acute rejection. Bone scintigraphy with (99m-Tc) pyrophosphate confirmed diagnosis. Symptoms spontaneously improved in 3–4 months in two patients and pain gradually resolved 6 months spontaneously or after prednisone in the other two patients.
Stamatoullas A., Ferrant A., and Manicourt D. (1993) [26]	Bone marrow transplantation (BMT), immunosuppression, chemotherapy, radiotherapy	Three patients (two men and one woman) with RSD of lower limbs. Painful feet and ankles, difficulty in standing-up or to walking during the early post-transplant period. Bone scintigraphy confirmed diagnosis of RSD. Two patients were unsuccessfully treated with calcitonin (50 U/day for 5 days), replaced by pamidronate (30 mg/d) for 5 days that was effective. Calcitonin (50 U/day for 5 days) improved symptoms in one patient.
Graham L.E. et al. (2002) [27]	Mastectomy in patients with chronic mastalgia unresponsive to all medical treatment	Two women with CRPS I of arm and hand. Pain (in one case, 4-year history of pain in left arm from the operation, and in the other case, after 4 weeks from the bilateral mastectomy operation), stiffness, reduction in articular mobility, hyperalgesia, edema and increased skin temperature. Diagnosis of CRPS was made after plain x-rays, investigations (including inflammatory indices) and in one case, after whole-body isotope bone scan. No improvements in the symptoms after intensive physiotherapy, intravenous infusions of pamidronate, and (in one case) despite two stellate ganglion nerve blocks and localized injections of botulinum toxin.
Chen Y.L. et al. (2014) [28]	Nuss procedure for correcting pectus excavatum	A 22-year-old man with CRPS I of right arm. Persistent pain, hyperalgesia, weakness, edema and color and temperature changes on right upper extremity developed 2 weeks after the operation. A three-phase bone scan showed increased uptake in the right elbow joint in the bone phase and slightly increased tracer distribution in the right elbow region during the arterial and blood-pooling phase. Patient was treated with intensive rehabilitation with improvement of the degree of pain, weakness and edema.

## Data Availability

The data that support the findings of this study are available from the corresponding author upon reasonable request.
